# The effect of dose escalation on gastric toxicity when treating lower oesophageal tumours: a radiobiological investigation

**DOI:** 10.1186/s13014-015-0537-y

**Published:** 2015-11-19

**Authors:** Rhys Carrington, John Staffurth, Samantha Warren, Mike Partridge, Chris Hurt, Emiliano Spezi, Sarah Gwynne, Maria A. Hawkins, Thomas Crosby

**Affiliations:** School of Medicine, Cardiff University, Cardiff, UK; Department of Clinical Oncology, Velindre Cancer Centre, Cardiff, UK; CRUK MRC Oxford Institute for Radiation Oncology Gray Laboratories, University of Oxford, Old Road Campus Research Building, Oxford, UK; Wales Cancer Trials Unit, Cardiff University, Cardiff, UK; School of Engineering, Cardiff University, Cardiff, UK; South West Wales Cancer Centre, Swansea, UK

## Abstract

**Purpose:**

Using radiobiological modelling to estimate normal tissue toxicity, this study investigates the effects of dose escalation for concurrent chemoradiation therapy (CRT) in lower third oesophageal tumours on the stomach.

**Methods and materials:**

10 patients with lower third oesophageal cancer were selected from the SCOPE 1 database (ISCRT47718479) with a mean planning target volume (PTV) of 348 cm^3^. The original 3D conformal plans (50Gy_3D_) were compared to newly created RapidArc plans of 50Gy_RA_ and 60Gy_RA_, the latter using a simultaneous integrated boost (SIB) technique using a boost volume, PTV2. Dose-volume metrics and estimates of normal tissue complication probability (NTCP) were compared.

**Results:**

There was a significant increase in NTCP of the stomach wall when moving from the 50Gy_RA_ to the 60Gy_RA_ plans (11–17 %, Wilcoxon signed rank test, *p* = 0.01). There was a strong correlation between the NTCP values of the stomach wall and the volume of the stomach wall/PTV 1 and stomach wall/PTV2 overlap structures (*R* = 0.80 and *R* = 0.82 respectively) for the 60Gy_RA_ plans.

**Conclusion:**

Radiobiological modelling suggests that increasing the prescribed dose to 60Gy may be associated with a significantly increased risk of toxicity to the stomach. It is recommended that stomach toxicity be closely monitored when treating patients with lower third oesophageal tumours with 60Gy.

## Introduction

The incidence of lower third oesophagus tumours are increasing in most Western populations [1] and it is becoming increasingly clear that chemo-radiotherapy (CRT) is now a valid alternative to surgical resection in the treatment of both oesophageal and gastroesophageal junction (GEJ) cancer [2] & [3]. It has been shown that a combined approach results in a significantly higher overall survival rate compared to either chemotherapy or radiotherapy alone [4] & [5]. However, local in-field recurrence is still the main reason of treatment failure [6] following definitive CRT, with >75 % of these occurring within the gross tumour volume (GTV) when the standard radiation dose of ≈ 50Gy is delivered. Indeed, local recurrence also contributes towards a worse prognosis in GEJ carcinoma [3].

In theory, a higher radiation dose delivered to the tumour should result in higher local control rate. However it is only with the recent technological advances in radiotherapy (RT) planning and delivery that the ability to deliver increased dose to the tumour whilst minimising dose to normal, healthy tissue and organs at risk (OAR) is becoming possible [7]. Increased tumour control probability (TCP) should therefore be achievable by increasing the standard dose prescription beyond ≈ 50Gy. A retrospective study by Zhang et al. [8] found that there was significantly higher overall survival in their patient cohort if the patient was treated in a high dose group (>51Gy) or a low dose group (<51Gy), whilst Geh et al. found there was a dose–response relationship between increasing prescribed radiotherapy dose and pathological complete response [9]. Bedford et al. [10] also found that conformal techniques offered the potential of a 5–10Gy increase in dose delivered to the GTV up to 60Gy with acceptable increases in toxicity.

The organs most at risk when planning oesophageal radiotherapy treatment, and for which the most stringent dose constraints are usually applied are the heart, lungs and spinal cord. Oesophageal cancer cases will therefore be planned according to a combination of the achievable dose coverage of the planning treatment volume (PTV) and the meeting of dose constraints for these organs. The SCOPE 1 study has shown low rates of acute and late toxicity with CRT using 4 cycles of cisplatin and capecitabine, with cycles three and four given concurrently with 50 Gy in 25 fractions of radiotherapy [11]. However, the 24 week failure free survival was significantly better in the CRT only arm than the CRT plus cetuximab arm (76 · 9 % (90 % confidence interval 69 · 7–83 · 0) vs 66 · 4 %, (58 · 6–73 · 6)) and cetuximab will therefore not be carried forward in future clinical trials. Work by this group in preparation for the forthcoming SCOPE 2 trial [12] has shown that dose escalation to 62.5Gy in mid oesophageal patients is feasible, with the additional dose able to be delivered without exceeding the OAR dose constraints in 75 % of patients. However, dose escalation has not yet been studied in lower oesophageal cancers, when the added proximity of the relatively radiosensitive stomach provides an added planning challenge [13]. With the role of radiotherapy dose escalation identified as a research priority [14] for improving outcomes, it is important to quantify the increased risk that this may pose in sites such as the lower oesophagus where clinical evidence for dose-toxicity correlation for adjacent organs (such as stomach) is lacking. This planning study therefore aims to investigate the feasibility of lower oesophageal dose escalation with a focus on toxicity to the stomach.

## Methods and materials

10 patients with tumours in the lower region (centre of tumour at 32–40 cm from back of teeth measured via Endoscopic ultrasound (EUS)) were selected at random from both arms of the SCOPE 1 database and their classification as lower region tumours confirmed visually. SCOPE1 has been ethically approved by the Research Ethics Committee for Wales and has approval from the Medicines and Health Care Product Regulatory Agency to be conducted in the UK. The subset had a range of planning target volumes (PTV1) from 219 to 484 cm^3^ and a mean volume of 348 cm^3^, similar to that of the entire SCOPE 1 cohort (mean 327 cm^3^). The GTVs and OARs outlined as per the SCOPE protocol were re-used.

PTV 1 is grown by adding 1 cm isotropcially to the clinical treatment volume (CTV), itself grown by adding 1 cm radially and 2 cm superiorly and inferiorly (along axis of oesophagus) to the GTV and may include the stomach mucosa at the inferior limit. For the purpose of this specific study and the use of the simultaneous integrated boost (SIB) technique for dose escalation, additional structures were also created. A PTV2 (boost volume) was created for the dose escalated plans by adding an isotropic 0.5 cm margin to the GTV, supported by a study by Hawkins et al. [15] and reflecting the technique in the SCOPE 2 trial where margins will not be adjusted dependent on tumour position [12]. The protocol did not address stomach filling or any dose constraints for that organ specifically. There were no constraints or protocol concerning the filling state of the stomach in the SCOPE 1 trial and therefore for the patients in this study. The stomach was contoured as (a) whole organ and (b) stomach wall. The stomach wall volume was generated by creating a ring like structure encompassing the outer 5 mm of the whole stomach outline. This has been shown to provide a satisfactory approximation of stomach wall thickness [16] & [17]. In addition, the stomach and stomach wall structures were divided into the volume that was within PTV1 (Stomach-In and StomachWall-In) and outside PTV1 (Stomach-Out and StomachWall-Out). Specific dose constraints were given for each for the SIB plans (Table 1) based on the recommendations of the Quantitative Analyses of Normal Tissue Effects in the Clinic (QUANTEC) paper for dose volume effects in the stomach and small bowel [18]. An SIB dose of 60Gy in 25 fractions was considered to be clinically meaningful and is being taken forward within an ongoing prospective dose escalation trial (SCOPE 2).

**Table 1:** Dose constraints for radiotherapy plans

Dose Constraints	
Dose-volume constraints	
PTV1 (50 Gy)	V_95%_ (47.5 Gy) > 95 %
	D_max_ (0.1 cc) < 107 % (53.5 Gy)
PTV2 (60 Gy)	V_95%_ (57 Gy) > 95 %
	D_max_ (0.1 cc) < 107 % (64.2 Gy)
Lung	Mean dose < 20 Gy
	V_20Gy_ < 25 %
Heart	Mean dose < 25 Gy
	V_30Gy_ < 45 %^a^
	V_40Gy_ < 30 %^b^
CordPRV	D_max_ (0.1 cc) < 40 Gy (45 Gy permitted)
Liver	V_30Gy_ < 60 %
Individual Kidneys	V_20Gy_ < 25 %
StomachIn^c^	Max dose < 60Gy
StomachOut^c^	Max dose < 45Gy

^a^Applies only to 50Gy_RA_ and 60Gy_RA_ plans

^b^Applies only to 50Gy_3D_ plans

^c^Applies only to 60Gy_RA_ plans

**Fig. 1:**
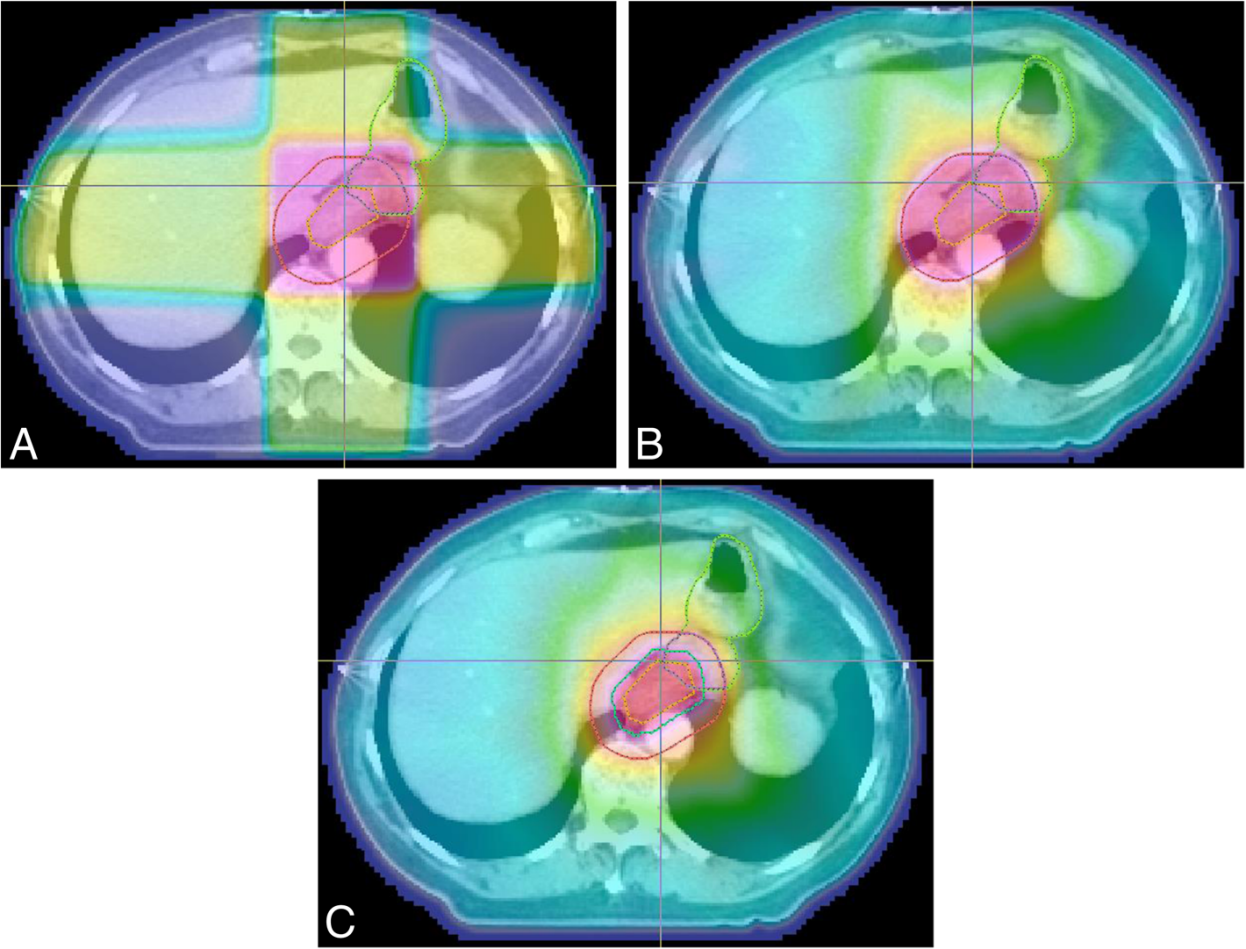
**a** 50Gy_3D_ plan with GTV, PTV and stomach outline. **b** 50Gy_RA_ plan with GTV, PTV and stomach outline. **c** 60Gy_RA_ plan with GTV, PTV2, PTV and stomach outline. Outlines: GTV– dashed orange, PTV– dashed red, PTV2– dashed blue, Stomach– dashed green

All treatment planning was undertaken in Eclipse version 10 (Varian, Palo Alto CA). The original 3D conformal plans were imported in DICOM format and the doses recalculated using the AAA algorithm with a 2.5 mm grid. RapidArc (RA) plans were generated using 2 arcs of 360^0^, clockwise and counter-clockwise with a collimator rotation of ±10^0^. The 50Gy 3D conformal plans (50Gy_3D_) were then compared to 50Gy RapidArc plans (50Gy_RA_) and to plans with an additional simultaneously integrated boost of 60Gy to PTV2 (60Gy_RA_) (See Fig. 1). Dose constraints are listed in Table 1 and additional dose-volume metrics were calculated for each structure (Table 2). Patient 6 was originally planned using 50Gy_RA_ therefore a 50Gy_3D_ plan was not created in this case.

**Table 2:** Dose volume metrics for all radiotherapy plans

Comparison of dose-volume metrics, TCP and NTCP values						
	50Gy_3D_	50Gy_RA_	60Gy_RA_	Wilcoxon signed-rank test		
	Median (range)	Median (range)	Median (range)	50Gy_3D_–50Gy_RA_	50Gy_3D_–60Gy_RA_	50Gy_RA_–60Gy_RA_
PTV1						
V95%	98.2 (96.0–100)	99.1 (95.2–100)	97.0 (95.0–98.2)	Z = 0.53 (*p* = .57)	Z = 1.07 (*p* = .28)	Z = 1.36 (*p* = .17)
PTV2 (GTV + 0.5 cm)						
V95%			95.1 (92.4–97.4)			
TCP (%) Geh	38.7 (37.5–41.1)	37.8 (37.5–38.7)	50.9 (50.7–51.4)	Z = 2.11 (*p* = .04)	Z = 2.67 (*p* = .01)	Z = 2.81 (*p* = .01)
Lung						
Mean dose (Gy)	9.8 (6.0–11.1)	10.2 (5.8–14.3)	10.7 (6.4–15.2)	Z = 1.78 (*p* = .07)	Z = 2.40 (*p* = .02)	Z = 2.80 (*p* = .01)
V13Gy (%)	26.8 (20.0–35.9)	32.8 (15.1–51.6)	34.4 (18.0–54.2)	Z = 2.19 (*p* = .03)	Z = 2.55 (*p* = .01)	Z = 2.09 (*p* = .04)
V20Gy (%)	19.7 (12.3–24.3)	11.3 (4.6–17.4)	15.6 (6.5–23.4)	Z = 2.55 (*p* = .01)	Z = 1.72 (*p* = .09)	Z = 2.81 (*p* = .01)
NTCP (%) De Jaeger	5.1 (1.9–6.0)	4.3 (2.8–8.0)	4.7 (3.1–9.0)	Z = 1.49 (*p* = .14)	Z = 2.09 (*p* = .04)	Z = 2.80 (*p* = .01)
Heart						
Mean dose (Gy)	26.8 (13.9–31.2)	21.2 (14.6–23.6)	20.2 (16.4–23.2)	Z = 1.68 (*p* = .09)	Z = 1.58 (*p* = .11)	Z = 0.15 (*p* = .88)
V30Gy (%)	55.1 (9.7–67.9)	17.2 (8.2–25.3)	18.7 (10.3–22.6)	Z = 2.67 (*p* = .01)	Z = 2.55 (*p* = .01)	Z = 0.87 (*p* = .39)
V40Gy (%)	16.2 (5.9–24.5)	10.1 (4.5–14.8)	10.6 (5.6–13.6)	Z = 2.67 (*p* = .01)	Z = 2.67 (*p* = .01)	Z = 1.58 (*p* = .11)
NTCP (%) Gagliardi	8.9 (3.1–12.8)	4.9 (2.2–7.3)	6.1 (2.9–7.9)	Z = 1.90 (*p* = .06)	Z = 1.38 (*p* = .17)	Z = 2.80 (*p* = .01)
Stomach						
Mean dose (Gy)	29.8 (5.5–44.2)	24.1 (5.4–40.4)	23 (6.5–36.1)	Z = 1.17 (*p* = .24)	Z = 0.97 (*p* = .33)	Z = 1.60 (*p* = .11)
Max dose (Gy)	52.6 (49.6–53.4)	51.9 (42.4–52.9)	60.9 (51.6–61.6)	Z = 0.83 (*p* = .41)	Z = 2.61 (*p* = .01)	Z = 2.81 (*p* = .01)
V45 (cc)	47.3 (7.3–80.4)	32.8 (0–49.8)	34.3 (5.4–25.4)	Z = 2.60 (*p* = .01)	Z = 2.50 (*p* = .01)	Z = 0.36 (*p* = .72)
V50 (cc)	31.5 (0–23.4)	17.7 (0–14.8)	21.4 (2.2–19.2)	Z = 2.31 (*p* = .02)	Z = 1.78 (*p* = .07)	Z = 1.27 (*p* = .20)
StomachIn max dose (Gy)	52.6 (49.6–53.4)	51.9 (42.4–52.9)	60.9 (51.6–61.6)	Z = 0.77 (*p* = .44)	Z = 2.61 (*p* = .01)	Z = 2.81 (*p* = .01)
StomachOut max dose (Gy)	51.4 (49.4–53.1)	44.4 (36.6–43.6)	44.8 (42.3–46.1)	Z = 1.76 (*p* = .07)	Z = 1.79 (*p* = .07)	Z = 0.14 (*p* = .88)
NTCP (%) Burman	0.6 (0–2.5)	0.2 (0–1.3)	0.3 (0–3.4)	Z = 2.38 (*p* = .02)	Z = 0.35 (*p* = .73)	Z = 2.03 (*p* = .04)
Stomach wall						
Mean dose (Gy)	29.5 (8.2–42.6)	22.9 (7.9–38.7)	22.4 (9.1–35.0)	Z = 0.97 (*p* = .33)	Z = 0.76 (*p* = .45)	Z = 0.87 (*p* = .39)
Max dose (Gy)	52.6 (49.6–53.4)	51.9 (43.4–52.9)	61 (51.6–61.6)	Z = 0.77 (*p* = .44)	Z = 2.55 (*p* = .01)	Z = 2.81 (*p* = .01)
V45 (cc)	28 (6.2–39.9)	17.9 (0–26.9)	17.9 (5.4–25.4)	Z = 2.19 (*p* = .03)	Z = 2.19 (*p* = .03)	Z = 0.46 (*p* = .65)
V50 (cc)	15.8 (0–23.4)	9.1 (0–14.8)	9.2 (2.2–19.2)	Z = 2.31 (*p* = .02)	Z = 1.48 (*p* = .14)	Z = 1.28 (*p* = .20)
NTCP (%) Feng	17.4 (3.5–24.9)	11.1 (3.6–18.9)	17.5 (3.2–39.4)	Z = 1.72 (*p* = .09)	Z = 1.99 (*p* = .05)	Z = 2.70 (*p* = .01)
Cord PRV						
Dmax 0.1 cc (Gy)	36.9 (16.1–41.3)	31.1 (26.2–44.1)	34.9 (28.4–39.6)	Z = 0.47 (*p* = .64)	Z = 0.18 (*p* = .86)	Z = 1.67 (*p* = .10)

Radiobiological modelling of TCP was undertaken using the parameters derived by Geh et al. [9]. This multivariate logistics regression model was constructed using data from 26 pre-operative CRT trials in oesophageal cancer and was considered a good representative of the SCOPE 1 patient cohort. The TCP modelling was undertaken bin-wise in Microsoft Excel using and parameters by Geh et al. found in their original paper [9]. Differential dose-volume histograms (DVH) for each structure were calculated in CERR utilising Matlab scripts developed in-house [19] before being converted to relative DVHs in Microsoft Excel. TCP was calculated as:

$$ TCP(z)=\frac{ \exp\;(z)}{1\kern0.5em +\kern0.5em  \exp\;(z)} $$

where z = a_0_ + a_1_ total RT dose + a_2_ total RT dose × dose per fraction + a_3_ duration + a_4_ age + a_5_ 5FU dose + a_6_ cisplatin dose. The α/β was 4.9Gy.

Normal tissue complication probability (NTCP) modelling was carried out in Eclipse Biological Evaluation module using the whole heart volume model of Gagliardi et al. [20] and for the lung using the model parameters from De Jaeger et al. [21], which predicts a radiation pneumonitis (RP) of grade 2 or higher. NTCP models for the stomach are limited therefore modelling was carried out using those judged to be most relevant. The whole stomach was modelled using parameters derived by Burman et al. [22] with the endpoint being ulceration, whilst the stomach wall parameters were derived by Feng et al. [23], modelling the probability of ≥3 grade gastric bleeding.

Data were analysed using the SPSS statistics package version 20.0.0 (IBM), and results are reported as median (range) values. Both the Z-score and the *P*-Values were calculated.

## Results

Table 2 reports the dose-volume metrics and the results of the Wilcoxon signed rank test for all radiotherapy plans. Adequate target dose coverage was possible for all patients in all treatment modalities when considering the coverage of PTV1 (Table 2). 4 patients failed to meet the minimum coverage of PTV2 with the minimum coverage being 92.4 %. All OAR dose for the heart and lung were met for all patients for all treatment plans. 6 patients failed to meet the Stomach-In constraint and 1 failed to meet the Stomach-Out constraint for the 60Gy_RA_ plans. All other dose constraints in Table 1 were met.

There was a mean decrease 1.0 % (−3.0 %, 0.6 %) in TCP from the 50Gy_3D_ to the 50Gy_RA_ plans, a mean increase of 12.0 % (9.9 %, 13.6 %) in TCP from the 50Gy_3D_ plans to the 60Gy_RA_ plans and a mean increase of 13.0 % (12.4 %, 13.4 %) in TCP from the 50Gy_RA_ plans to the 60Gy_RA_ plans. For NTCP there was a mean decrease of 3.4 % (−6.3 %, 0 %) for the heart from the 50Gy_3D_ to the 50Gy_RA_ plans, a mean decrease of 2.2 % (−4.9 %, 2.0 %) from the 50Gy_3D_ to the 60Gy_RA_ plans and a mean increase of 1.2 % (0.5 %, 2.0 %) in NTCP for the heart from the 50Gy_RA_ to the 60Gy_RA_ plans. For lung there was a mean increase of 0.4 % (−0.8 %, 2.2 %) in NTCP from the 50Gy_3D_ to the 50Gy_RA_ plans, a mean increase of 1.0 % (−0.6 %, 3.2 %) from 50Gy_3D_ to 60Gy_RA_, and a mean increase of 0.6 % (0.1 %, 1.2 %) from the 50Gy_RA_ to the 60Gy_RA_ plans.

**Fig. 2:**
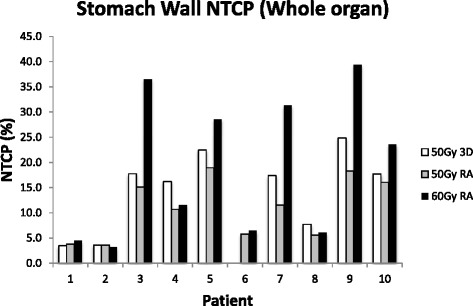
NTCP for whole stomach wall for 50Gy_3D_, 50Gy_3D_ and 60Gy_RA_ radiotherapy plans

For the stomach and stomach wall the variation in NTCP between patients was considerable. Patients 1, 2, 6 & 8 all had stomach NTCP values <0.03 % for all treatment plans whilst the largest value was 3.4 % for a patient planned using the 60Gy_RA_ technique. The stomach wall model, which models a different endpoint, showed considerably larger absolute values of NTCP, the largest being 39.4 % for a patient treated with the 60Gy_RA_ plan. Across the whole study, there was a mean decrease in stomach wall NTCP of 3.1 % (−6.5, 0 %) from the 50Gy_3D_ plans to the 50Gy_RA_ plans, a mean increase of 5.9 % (−4.7, 18.7 %) in NTCP from the 50Gy_3D_ to the 60Gy_RA_ plans and a mean increase of 8.2 % (−0.4, 21.3 %) in NTCP from the 50Gy_RA_ to the 60Gy_RA_ plans (NTCP values see Fig. 2).

**Fig. 3:**
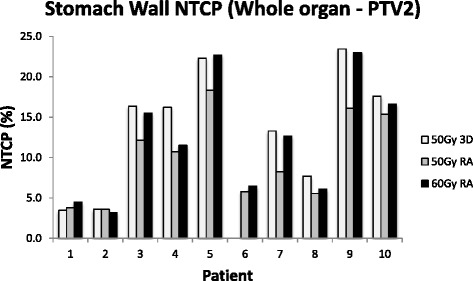
NTCP for stomach wall minus PTV2 for 50Gy_3D_, 50Gy_3D_ and 60Gy_RA_ radiotherapy plans

When the NTCP modelling is restricted to the volume outside the boost volume (PTV2), there was in general a smaller difference between the NTCP values between plans. In this case there was a mean decrease of 3.4 % (−7.4 %, 0.3 %) from the 50Gy_3D_ to the 50Gy_RA_ plans, a mean decrease of 0.9 % (−4.7 %, 1.0 %) in NTCP from the 50Gy_3D_ to the 60Gy_RA_ plans, and a mean increase of 2.3 % (−0.4 %, 6.9 %) in NTCP from the 50Gy_RA_ to the 60Gy_RA_ plans (Fig. 3).

Table 3 shows the Pearson correlation coefficients between the Stomach and Stomach Wall volumes and associated dose metrics. It can be seen how the strongest correlations are between the stomach wall volumes in each plan and the mean does received by those volumes (0.63, 0.66 and 0.66 for the 50Gy_3D_, 50Gy_RA_ and 60Gy_RA_ respectively).

**Table 3:** Pearson correlation coefficients between stomach, stomach wall volumes and dose metrics

	Pearson Coefficient		
	50Gy3D	50GyRA	60GyRA
Stomach Volume - Stomach Mean Dose	0.35	0.60	0.61
Stomach Volume - Stomach Max Dose	−0.19	0.12	0.55
Stomach Volume - Stomach V45	0.16	0.08	−0.02
Stomach Volume - Stomach V50	0.11	0.05	−0.04
Stomach Wall Volume - Stomach Wall Mean Dose	0.63	0.66	0.66
Stomach Wall Volume - Stomach Wall Max Dose	−0.12	0.32	0.68
Stomach Wall Volume - Stomach Wall V45	0.23	0.21	0.12
Stomach Wall Volume - Stomach Wall V50	0.38	0.22	0.04

**Fig. 4:**
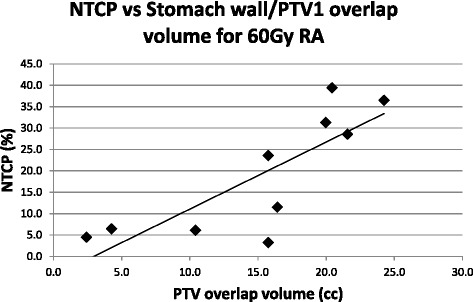
NTCP vs whole stomach wall/PTV1 overlap structure volume for 60Gy_RA_ radiotherapy plans

Six patients had an overlap between the GTV and PTV2 and Stomach Wall structure whilst all patients had an overlap between the PTV1 and Stomach Wall structures. There was a strong correlation between the NTCP value and the Stomach Wall structure/PTV1 overlap structure volume for all treatment plans (Pearson’s *R* = 0.80, 0.77 and 0.77 for the 60Gy_RA_, 50Gy_RA_ and 50Gy_3D_ plans respectively). Fig. 4 shows the correlation between NTCP and the Stomach Wall/PTV1 overlap structure volume for the 60Gy_RA_ plans.

**Fig. 5:**
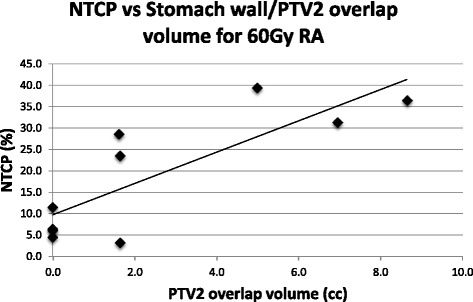
NTCP vs whole stomach wall/PTV2 overlap structure volume for 60Gy_RA_ radiotherapy plans

There was also a strong correlation between the NTCP value and the Stomach Wall/PTV2 overlap structure volume for the 60Gy_RA_ plan (*R* = 0.82) (Fig. 5).

## Discussion

This study has shown that using the SIB technique it is possible to deliver a dose of 60Gy to the tumour whilst adhering to all standard OAR dose constraints for lower oesophagus tumours.

It is acknowledged that the TCP model used in this investigation does not account for Cetuximab administration, however Cetuximab will not be administered in the SCOPE 2 trial at which this study is aimed. A strength of the TCP model proposed by Geh et al. is that it combines a diverse range of trials, and it was therefore considered the most appropriate to use here. It has been shown that there is a small reduction (<1 %) in TCP when comparing the 50Gy_3D_ plans to the 50Gy_RA_ plans. There was a higher lung mean V13Gy, but reduced V20Gy, Heart V30 /40Gy, Stomach V45/50 cc and Stomach Wall V45/50 cc. When comparing the 50Gy_RA_ to the 60Gy_RA_ plans there was a significant increase in TCP but also an increase in the mean dose parameter for the lung (See Table 2). There was a significant increase in mean TCP (≈12) going from the 50Gy_3D_ to the 60Gy_RA_ plan. Comparing 50Gy_3D_ and 50Gy_RA,_ there was a statistically significant increase in lung V13Gy, which can be explained by the low dose wash associated with RapidArc type treatment plans, however V20Gy reduced and mean lung NTCP was reduced from 5.1 % to 4.3 %. There was a significant decrease in the heart V30/40Gy values. Although this did not result in a significant decrease in NTCP between the two planning methods in this study, this agrees with results from our previous work on mid-oesophageal cancer patients [12].

Moving from the 50Gy_RA_ to the 60Gy_RA_ plans, although the NTCP values for the heart and lung were lower than those found in our previous study on mid oesophageal cancer patients as would be expected, there was still a similar modest increase in heart and lung toxicities when using the boost technique [12]. This also agrees with the recently published study by Roeder et al. who delivered 60Gy to patients with oesophageal cancer using a SIB technique and found acceptable acute and late overall toxicity to the lung and heart [24]. However, when treating lower oesophageal tumours there is the added complication of having the stomach adjacent to the treatment volume. The involvement of the group in a proposed randomized clinical trial investigating dose escalation (SCOPE 2) therefore led to this study, which is the first to specifically investigate the effect of dose escalation in lower oesophageal tumours on the stomach using radiobiological modelling. It is acknowledged that biological modelling and the resulting outcomes are extremely dependent on the model parameters used as well as how they are applied. As a result, we used two models for the stomach and applied them to both to the structure as a whole and inside and outside the PTV. The model for the stomach wall by Feng et al. [23] was found to predict a higher rate of toxicity than that for the whole stomach, most likely the result of the different endpoints of gastric bleeding and ulceration being modelled respectively. Max dose constraints of 45Gy and 60Gy were applied to the stomach outside (Stomach-Out) and inside (Stomach-In) the PTV respectively for the 60Gy_RA_ plans. The NTCP results for the 60Gy_RA_ when modelling the volume outside the PTV were similar to those of the 50Gy_RA_ and 50Gy_3D_ plans (Max NTCP of 23.0 % and 23.4 % for the 60Gy_RA_ and 50Gy_3D_ plans respectively), suggesting that dose escalation may not pose any more risk to normal stomach than 3D conformal radiotherapy (Fig. 3). However, when considering the stomach wall structure as a whole it was found that there was up to 20 % increase in NTCP when using the dose escalation plan compared to the 50Gy_RA_ plan. This value however could be considered as being the worse case scenario, as it is acknowledged that stomach movement and filling over the course of treatment may blur out any dose hot spots. The analysis of any accompanying Cone Beam CT data of these patients would help quantify this movement however this data was unavailable. Any NTCP value is also by nature calculated from a model that is open to interpretation therefore should only be used to give an approximate risk. It is fully acknowledged that radiobiological modelling inherently has limitations that limit its accuracy. Specifically in the case of this study, there is a lack of both clinical outcome data and radiobiological models for stomach toxicity when prescribing a dose >50Gy. However the model used was deemed to be the most suitable in this instance. The application of radiobiological modelling to partial organ irradiation is also a contentious one that may affect the results. However the purpose of this study was not to give definitive values of stomach toxicity, but to investigate and inform of the potential relative risks involved in dose escalation of lower esophageous tumours both in a forthcoming trial and in clinical practice.

We have shown that there is a strong correlation in NTCP with the volume of overlap between the stomach wall with both PTV1 and the high dose region PTV2. When more clinical data is available it may become apparent that safe delivery of the 60Gy SIB is dependent on this volume of the overlap, which could potentially be reduced by reducing the treatment margins for individual patients using techniques such as 4DCT, gating and breath hold protocols. However it has been reported that the inter-patient motion of oesophageal tumours is highly variable [25] and that even the use of 4DCT may not even fully account for organ motion in between fractions [26]. Nakamura et aldiscuss how large variations in stomach volume may have a detrimental effect on dose escalation when treating pancreatic cancer, despite using a breath hold technique [27]. The impact of variation in gas in the stomach on dose distribution should also be considered. For example, Kumagai et al. found that dose conformation to the CTV was degraded due to bowel gas movement when treating pancreatic cancer using carbon ion beams [28] and consequently may also be applicable when using photon beams. Bouchard et al. also found that changes in stomach filling resulted in the boost target being missed when treating GEJ tumours with IMRT-SIB [29]. A move to reduction in population-based margins from those used in the SCOPE 1 and SCOPE 2 trials, rather than on an individual basis, may therefore increase the risk of failure to control the disease. The margins used in this investigation were taken from the SCOPE 2 protocol therefore give an approximation of results from a forthcoming nationwide trial, taking into account the inherent errors in radiobiological modelling.

Concerning the impact of stomach filling, as there was no stomach filling protocol for the SCOPE 1 trial an area for further work would; be to investigate what impact, if any, the inclusion of a filling or breath hold protocol would have on stomach toxicity and dose distribution when treating lower oesophageal tumours. However this is beyond the scope of this current work as it would require either the incorporation of a protocol into a clinical trial made available for analysis or a retrospective analysis of patients who underwent an appropriate strategy prior to treatment.

An inclusion criteria for the SCOPE 1 trial was that patients were to have histologically confirmed carcinoma of the oesophagus with no more than 2 cm of mucosal tumour extension into the stomach. As this patient group is likely to be included in the SCOPE 2 trial, this study’s findings mean it is likely that it be advised in the radiotherapy protocol that these patients be treated with caution until the safety of this dose escalation method is clearly defined within the SCOPE 2 trial.

The results of this study also suggest that the maximum prescribed dose achievable for each patient may be dependent on the volume of the stomach overlap with the treatment volume. Further work will therefore include modelling individualised dose prescription.

## Conclusion

Radiobiological modelling suggests that increasing the prescribed dose to 60Gy may be associated with a significantly increased risk of toxicity to the stomach within the boost volume. The results of this study also suggest that the maximum prescribed dose safely achievable for each patient in the future may be dependent on the volume of the stomach within the treatment volume. It is recommended that stomach toxicity be closely monitored prospectively when treating patients with lower oesophageal tumours in the forthcoming SCOPE 2 trial.

## Abbreviations

CRT: Chemo-radiotherapy
CTV: Clinical Treatment Volume
DVH: Dose Volume Histogram
EUS: Endoscopic Ultrasound
GEJ: Gastroesophageal Junction
GTV: Gross Tumour Volume
NTCP: Normal Tissue Complication Probability
OAR: Organ At Risk
PRV: Planning Risk Volume
PTV: Planning Treatment Volume
RA: RapidArc
RT: Radiotherapy
SIB: Simultaneous Integrated Boost
TCP: Tumour Control Probability
